# Virus Identification in Unknown Tropical Febrile Illness Cases Using Deep Sequencing

**DOI:** 10.1371/journal.pntd.0001485

**Published:** 2012-02-07

**Authors:** Nathan L. Yozwiak, Peter Skewes-Cox, Mark D. Stenglein, Angel Balmaseda, Eva Harris, Joseph L. DeRisi

**Affiliations:** 1 Division of Infectious Diseases and Vaccinology, School of Public Health, University of California, Berkeley, California, United States of America; 2 Biological and Medical Informatics Program, University of California San Francisco, San Francisco, California, United States of America; 3 Howard Hughes Medical Institute, University of California San Francisco, San Francisco, California, United States of America; 4 Department of Biochemistry and Biophysics, University of California San Francisco, San Francisco, California, United States of America; 5 Departamento de Virología, Centro Nacional de Diagnóstico y Referencia, Ministerio de Salud, Managua, Nicaragua; 6 Department of Medicine, University of California San Francisco, San Francisco, California, United States of America; Texas Biomedical Research Institute, United States of America

## Abstract

Dengue virus is an emerging infectious agent that infects an estimated 50–100 million people annually worldwide, yet current diagnostic practices cannot detect an etiologic pathogen in ∼40% of dengue-like illnesses. Metagenomic approaches to pathogen detection, such as viral microarrays and deep sequencing, are promising tools to address emerging and non-diagnosable disease challenges. In this study, we used the Virochip microarray and deep sequencing to characterize the spectrum of viruses present in human sera from 123 Nicaraguan patients presenting with dengue-like symptoms but testing negative for dengue virus. We utilized a barcoding strategy to simultaneously deep sequence multiple serum specimens, generating on average over 1 million reads per sample. We then implemented a stepwise bioinformatic filtering pipeline to remove the majority of human and low-quality sequences to improve the speed and accuracy of subsequent unbiased database searches. By deep sequencing, we were able to detect virus sequence in 37% (45/123) of previously negative cases. These included 13 cases with Human Herpesvirus 6 sequences. Other samples contained sequences with similarity to sequences from viruses in the *Herpesviridae*, *Flaviviridae*, *Circoviridae*, *Anelloviridae*, *Asfarviridae*, and *Parvoviridae* families. In some cases, the putative viral sequences were virtually identical to known viruses, and in others they diverged, suggesting that they may derive from novel viruses. These results demonstrate the utility of unbiased metagenomic approaches in the detection of known and divergent viruses in the study of tropical febrile illness.

## Author Summary

Dengue virus infection is a global health concern, affecting as many as 100 million people annually worldwide. A critical first step to proper treatment and control of any virus infection is a correct diagnosis. Traditional diagnostic tests for viruses depend on amplification of conserved portions of the viral genome, detection of the binding of antibodies to viral proteins, or replication of the virus in cell cultures. These methods have a major shortcoming: they are unable to detect divergent or novel viruses for which *a priori* sequence, serological, or cellular tropism information is not known. In our study, we use two approaches, microarrays and deep sequencing, to virus identification that are less susceptible to such shortcomings. We used these unbiased tools to search for viruses in blood collected from Nicaraguan children with clinical symptoms indicating dengue virus infection, but for whom current dengue virus detection assays yielded negative results. We were able to identify both known and divergent viruses in about one third of previously negative samples, demonstrating the utility of these approaches to detect viruses in cases of unknown dengue-like illness.

## Introduction

Viral infections pose a significant global health burden, especially in the developing world where most infectious disease deaths occur in children and are commonly due to preventable or treatable agents. Effective diagnostic and surveillance tools are crucial for reducing disability-adjusted-life-years (DALYs) due to infectious agents and for bolstering elimination and treatment programs [1]. Previously unrecognized and novel pathogens continually emerge due to globalization, climate change, and environmental encroachment, and pose important diagnostic challenges [2], [3].

Dengue virus (DENV) infection is the most common arthropod-borne viral disease of humans, with an estimated 50–100 million clinical infections occurring annually worldwide [4]. DENV infection manifests clinically as dengue fever or the more severe dengue hemorrhagic fever/dengue shock syndrome (DHF/DSS) [4]. The increased spread of dengue virus and its mosquito vectors in many subtropical regions over the past several decades, especially in Latin America and Asia [5], highlights the need for additional methods of dengue virus surveillance. Diagnosing dengue relies on detecting viral nucleic acid or antigens in the blood or confirming the presence of anti-DENV IgM and IgG antibodies and therefore traditionally depends on RT-PCR, ELISA, and viral cell culture methods [5]–[7]. Dengue diagnostics are of crucial importance due to its broad spectrum of clinical presentations, global emergence and spread, unique disease epidemiology, and possible clinical relation to other as-yet unknown tropical febrile pathogens.

Traditional viral detection methods, such as serology, virus isolation, and PCR, are optimized for the detection of known agents [2]. However, novel and highly divergent viruses are not easily detected by approaches that rely on *a priori* sequence, antigen, or cell tropism knowledge. PCR-based assays that employ degenerate primers may successfully target conserved regions within related virus groups, but unlike bacteria, viruses lack universally conserved genetic regions, such as ribosomal RNA, that can be exploited to amplify all viruses [8].

Metagenomic analysis enables more systemic detection of both known and novel viral pathogens [9]–[12] and is approached through a variety of microarray and sequencing strategies [13], [14]. The Virochip is a pan-viral microarray platform that has been previously utilized in the detection and discovery of viruses from both human and animal samples [15]–[19]. Deep sequencing and shotgun sequencing of human clinical samples has been used for viral detection [20]–[23], novel virus discovery [24]–[27], and divergent virus genome recovery [28]. Viral metagenomic approaches have also been employed as a diagnostic supplement to pathogen detection as part of public health monitoring systems [22], but have been limited to shotgun sequencing of viral-enriched libraries and have yet to utilize deep sequencing data. Currently available sequencing platforms can generate millions to billions of sequencing reads per run, far exceeding large-scale shotgun sequencing [13]. Deep sequencing of clinical samples, in which hundreds of thousands to millions of sequencing reads are generated per sample, can be incorporated into stepwise virus detection pipelines [29]. Database searches using Basic Local Alignment Search Tool (BLAST) and other alignment tools [30] can be used to identify sequences in samples that correspond to known and novel viruses, including those present at low concentrations or deriving from viruses that may be too divergent to be detected with PCR or microarray methods. Deep sequencing represents an unbiased, highly sensitive method for identifying viral nucleic acid in clinical samples.

This study describes the use of the Virochip microarray and deep sequencing for the direct viral diagnosis of serum from cases of acute pediatric febrile illness in a tropical urban setting. Patient clinical data and serum samples were collected between 2005 and 2009 as part of an ongoing pediatric dengue study in Managua, Nicaragua [31]. Virochip and deep sequencing were performed on positive control samples and on 123 dengue virus-negative serum samples. Using these methods, viruses were detected in 45 of 123 (37%) previously negative samples. Sequences derived from known and apparently divergent viruses. The viruses identified in some of the cases are known to induce symptoms consistent with those observed, though the definitive causative agent of these infections remains to be determined.

## Methods

### Study Population

Acute serum samples were collected from suspected dengue cases at the Hospital Infantil Manuel de Jesús Rivera (HIMJR), the National Pediatric Reference Hospital in Managua, Nicaragua, after undergoing informed consent or the informed consent procedure. Patients were enrolled in the study if they presented with fever or history of fever less than 7 days and one or more of the following signs and symptoms: headache, arthralgia, myalgia, retro-orbital pain, positive tourniquet test, petechiae, or signs of bleeding. Patients with a defined diagnosis other than dengue, *e.g.* pneumonia, were excluded. Suspected dengue cases were tested for dengue virus (DENV) infection at the Centro Nacional de Diagnóstico y Referencia (CNDR) of the Nicaraguan Ministry of Health and were considered laboratory-confirmed if: 1) DENV was isolated, 2) DENV RNA was detected by reverse transcriptase-polymerase chain reaction (RT-PCR), 3) seroconversion was observed by IgM capture enzyme-linked immunosorbent assay (ELISA) of paired acute and convalescent sera, or 4) a ≥4-fold increase in DENV-specific antibodies was demonstrated by inhibition ELISA in paired acute and convalescent sera [32]. All patients were aged 6 months to 14 years and presented between August 2005 and January 2009.

Approximately one half of the suspected dengue cases testing negative by all four dengue diagnostic assays were included in the metagenomics analysis described here. 34 cases (pools 1–4, see below) corresponded to the subset of patients who presented within 4 days of symptom onset and who reported both fever or history of fever and rash. 89 of the samples (pool 5) were selected randomly from among the remaining samples. As positive controls, seven samples (pool 5) that had been clinically diagnosed as virus positive were included. The study protocol was reviewed and approved by the Institutional Review Boards (IRB) of the University of California, Berkeley, and of the Nicaraguan Ministry of Health.

### Metagenomic Library Preparation

Total nucleic acid from 140 µl of serum was extracted using the QIAamp Viral RNA Isolation Kit (Qiagen), which co-purifies RNA and DNA. End-tagged dsDNA libraries were created essentially as previously described [28]. RNA was reverse transcribed in reactions containing 1× reaction buffer, 5 mM dithiothreitol, 1.25 mM dNTPs, 20 pmoles primer (5′-CGC TCT TCC GAT CTN NNN NN-3′), 100 U Superscript III (Invitrogen), and ∼20 ng template. Following reverse transcription, Sequenase reaction buffer and 2 U of Sequenase DNA polymerase (Affymetrix) were added to samples for second strand synthesis. The Sequenase reactions were performed twice so that starting DNA templates would be converted into end-tagged library molecules. The resulting libraries were amplified by PCR using primer 5′-CGC TCT TCC GAT CT-3′. PCRs contained 1× reaction buffer, 2 µM primer, 0.25 mM dNTPs, 2 U Taq DNA polymerase, and 2 µl library template. Thermocycling conditions were 95°C for 2 min; 25 cycles of 95°C for 30 sec, 40°C for 30 sec, and 72°C for 1 minute, with a final extension of 5 minutes. These libraries were further processed for microarray hybridization and deep sequencing as described below.

For microarray hybridization, a fraction of each library was amplified by PCR as above but with a modified dNTP mixture including 5-(3-aminoallyl)-dUTP (Ambion) in lieu of 75% of the dTTP normally in the mixture. The resulting amino-allyl-containing DNA was purified using a DNA Clean and Concentrator-5 column (Zymo Research). The eluate was heat denatured at 95°C for 2 min, cooled briefly on ice, then fluorescently labeled in reactions containing 100 mM sodium bicarbonate pH 9, 10% DMSO, and 667 µM Cy3 mono NHS ester (GE Healthcare) for 1 hour at 25°C. Labeled DNA was purified using DNA-CC-5 columns and added to hybridization reactions containing 3×SSC, 25 mM HEPES pH 7.4, and 0.25% SDS. Hybridization mixtures were heated at 95°C for 2 minutes, applied to microarrays, and hybridized overnight at 65°C. Following hybridization, arrays were washed twice in 0.57× SSC and 0.028% SDS and twice in 0.057× SSC, then scanned on an Axon GenePix 4000B microarray scanner. Three analysis tools were used to analyze Virochip data: E-predict [33], Z-score analysis [34], and cluster analysis [35]. An array was deemed positive for a particular virus if the virus was identified by at least two of these methods. Virochip results were deposited in the NCBI GEO database (GEO accession series: GSE28142).

For deep sequencing, the Illumina paired-end adapter sequences were appended to library molecules using PCR, essentially as previously described [28]. Library generation primers (Table S1) were modified from adapter A and adapter B sequences (Illumina). Samples were reverse transcribed and libraries were created and amplified as described above for the Virochip. Library molecules of approximately 300 bp were purified on a 4% native polyacrylamide gel, ethanol precipitated, and PCR amplified for 17 additional cycles using a 22-nt-long primer consisting of the 3′-end of Illumina adapter A (primer 2) and the full-length 61-bp Illumina adapter B (primer 4) under the following conditions: 2 cycles of 94°C for 30 s, 40°C for 30 s, and 72°C for 1 min, followed by 15 cycles of 94°C for 30 s, 55°C for 30 s, and 72°C for 1 min. Amplicons generated with the correct adapter topology (one end with adapter A and the other with adapter B) were approximately 355 bp and were separated by polyacrylamide gel electrophoresis from adapter A/A and adapter B/B amplicons, which migrate differently (approximately 40 bp smaller or larger than the expected size). An additional 10 cycles of PCR were then performed using the full-length adapter sequences as primers (primers 3 and 4). Libraries were validated by Sanger sequencing before high throughput sequencing. Following validation, samples were combined into five pools for sequencing. For pools one through four reverse transcription primers included a three or four-nucleotide barcode sequence at the 3′-end. For pool five, barcodes were located internally in the adapter sequence. Each pool was sequenced on one lane of a flowcell on the Illumina Genome Analyzer II (pools 1–4) or HiSeq 2000 (pool 5). Pools' 1–4 molecules were sequenced as 67 nucleotide paired ends, and pool 5 molecules as 97 nucleotide paired ends. Paired-end sequencing was performed for several reasons: (1) to double the overall amount of data generated, (2) to double the amount of sequence information per molecule, and (3) to provide anchors from which additional sequence could be recovered by subsequent PCR.

### Virus Sequence Recovery

In some cases, PCR and Sanger sequencing was used to confirm Virochip and deep sequencing calls and to recover additional sequence. Primer sequences are listed in Table S1. PCR conditions were: 95°C for 2 minutes, 35 cycles of 95°C for 30 seconds, 50–60°C for 30 seconds (primer dependent), 72°C for 1 minute, and 72°C for 2 minutes. PCR products were size-selected on an agarose gel, purified with the Purelink gel extraction kit (Invitrogen), cloned, and Sanger sequenced.

### Virochip Sensitivity

Full-length poliovirus genomic RNA was transcribed from MluI-linearized plasmid prib(+)XpA using T7 RNA polymerase as previously described [36]. Poliovirus RNA was mixed with HeLa total RNA in a dilution series ranging from 10^−2^ to 10^−6^ poliovirus gRNA per HeLa RNA. Randomly-primed dsDNA libraries were prepared, hybridized to the Virochip, and analyzed as described above.

### Phylogenetic Analysis

Predicted circovirus-like replicase sequences were searched against the NCBI non-redundant protein database (BLASTx, E value 10^−2^). Aligning sequences were retrieved and consolidated using CD-HIT into a set of representative sequences [37] (CD-HIT version 4.5.4; parameters: -c 0.7). These sequences were aligned in Geneious [38] as a global alignment with free end gaps and trimmed to the 47 amino acid overlap shared by the two recovered sequences. A neighbor-joining tree was generated by Geneious Tree Builder [38].

### Deep Sequencing Bioinformatics

The initial FASTQ data from each pool's lane were binned by barcode. The barcode-split reads were trimmed of non-template deriving and potentially error-prone sequence: a randomly incorporated nucleotide (N), the barcode bases, and the sequence corresponding to the random hexamer, leaving 55 (pools 1, 2, and 4), 54 (pool 3), or 90 (pool 5) bases per read. The lowest complexity fraction was identified by sequences with LZW ratios (compressed size/uncompressed size) less than 0.45 [39]. Reads were aligned to the human genome (build hg18) first using BLAT [40] with the “–fastMap” flag, and after filtering, the remaining reads were aligned using BLAT without the flag. Paired reads for which at least one of the reads in the pair had at least 80% identity to the database were marked as human and removed from subsequent analyses. After removal of reads identified as human by BLAT, remaining reads were aligned and filtered by mapping to the human transcriptome using nucleotide BLAST (BLASTn version 2.2.21, word size 30, E value 10^−3^). Remaining reads were next aligned to the human genome using BLASTn (word size 30, E value 10^−3^), filtered, and again aligned to the human genome by BLASTn (word size 11, E value 10). After all human filtering, we reanalyzed the distribution of the complexity of reads and observed a relative enrichment of reads with LZW ratios lower than 0.54 (pools1–4) or 0.48 (pool5; different LZW ratio distributions are an inherent property of different read lengths), and those reads were removed from further analysis. To look for reads with viral homology, we searched the non-redundant nucleotide database (nt) using BLASTn (word size 20, E value 10^−3^). Reads that did not map to nt were aligned to the non-redundant protein database (nr) using translated BLAST (BLASTx, word size 4, E value 10).

### Virus Sequence Detection

In order to make specific virus-positive calls, we implemented a set of rules to minimize false positives while maintaining sensitivity. In order to reduce the number of false positive sequences that may share identity equally with both viral and non-viral genomes, we restricted our analysis to those queries whose best alignments were only to animal viral sequences. In a number of datasets, we detected human klassevirus 1, a virus identified and studied in our lab [26], human poliovirus, used in our Virochip sensitivity experiments, sequences from mosquito densoviruses, also studied in the lab, as well as Moloney murine leukemia virus (MMLV), the polymerase of which was used in the sequence library preparation. We believe these reads represent lab contaminants, and others studies that prepared sequence libraries in the same location have reported similar findings [41]. To account for these contaminants, positive calls were only made on viruses for which there were more supporting reads than there were reads to any known contaminant. Finally, in order to avoid making calls based on potentially spurious alignments, we considered only those viruses for which there were at least 10 reads supporting their presence.

## Results

### Virochip Analysis

We initially screened the serum samples with the Virochip pan viral detection microarray. This was done as a complement to the deep sequencing analysis and in order to compare the sensitivity of the two approaches. We included 7 blinded positive control samples that had been previously diagnosed in the clinic as being positive for DENV-2 (n = 4), DENV-1 (n = 1), or hepatitis A virus (HAV; n = 2). The Virochip successfully identified the correct virus in all of these positive controls, and in the case of the dengue virus positive samples, the correct serotype as well (Table 1). We also identified ten samples positive for torque teno virus (TTV).

**Table 1:** Summary of viruses identified in this study.

Patient code	Clinic virus ID	Virochip virus ID	Sequencing virus ID	Virus TaxID^a^	# virus reads	# initial reads	Fraction virus reads
187	DENV-2	DENV-2	Dengue virus 2	11060	4280	1.1E+06	3.9E−03
275	DENV-2	DENV-2	Dengue virus 2	11060	1511	1.6E+06	9.7E−04
282	DENV-2	DENV-2	Dengue virus 2	11060	699	1.6E+06	4.2E−04
266	DENV-2	DENV-2	Dengue virus 2	11060	135749	4.8E+06	2.8E−02
274	DENV-1	DENV-1	Dengue virus 1	11053	27	1.2E+06	2.3E−05
401^b^	HAV	HAV	Hepatitis A virus	12092	2164	1.8E+05	1.2E−02
401^b^	HAV	HAV	Hepatitis A virus	12092	4562	1.3E+06	3.5E−03
235	-	-	Human herpesvirus 6	10368	116	5.5E+06	2.1E−05
451	-	-	Human herpesvirus 6	10368	88	2.7E+06	3.2E−05
207	-	-	Human herpesvirus 6	10368	390	9.6E+06	4.1E−05
432	-	-	Human herpesvirus 6	10368	411	3.5E+06	1.2E−04
574	-	-	Human herpesvirus 6	10368	138	3.2E+06	4.4E−05
370	-	-	Human herpesvirus 6	10368	90	3.2E+06	2.9E−05
78	-	-	Human herpesvirus 6	10368	113	1.2E+06	9.8E−05
131	-	-	Human herpesvirus 6	10368	24	1.2E+06	2.0E−05
183	-	-	Human herpesvirus 6	10368	66	3.0E+06	2.2E−05
270	-	-	Human herpesvirus 6	10368	28	1.2E+06	2.4E−05
344	-	-	Human herpesvirus 6	10368	303	1.3E+06	2.2E−04
350	-	-	Human herpesvirus 6	10368	48	3.0E+06	1.6E−05
438	-	-	Human herpesvirus 6	10368	72	4.4E+06	1.6E−05
315	-	-	African swine fever virus	10497	42	1.9E+06	2.2E−05
382	-	-	Human herpesvirus 4	10376	44	9.6E+05	4.6E−05
387	-	-	GB virus C	54290	171	9.0E+06	1.9E−05
180	-	-	GB virus C	54290	42	8.0E+05	5.2E−05
161	-	-	Human parvovirus B19	10798	14	3.0E+06	4.7E−06
118	-	-	Circovirus-like genome RW-E	642255	177	7.4E+06	2.4E−05
323	-	-	Circovirus-like genome RW-E	642255	12	5.0E+06	2.4E−06
363	-	-	Circovirus-like genome RW-E	642255	17	1.9E+06	8.9E−06
371	-	-	Circovirus-like genome RW-E	642255	21	1.6E+06	1.3E−05
387	-	-	Circovirus-like genome RW-E	642255	92	9.0E+06	1.0E−05
355	-	-	Beak and feather disease virus	77856	12	2.1E+06	5.7E−06
345	-	-	Beak and feather disease virus	77856	62	2.2E+06	2.9E−05
315	-	-	Swan circovirus	459957	26	1.9E+06	1.4E−05
329	-	-	Gull circovirus	400121	14	2.2E+06	6.3E−06
321	-	-	Porcine circovirus 1	133704	30	4.6E+06	6.5E−06
375	-	-	Porcine circovirus 1	133704	53	3.8E+06	1.4E−05
377	-	-	Cyclovirus PK5034	742916	81	6.6E+06	1.2E−05
322	-	-	Cyclovirus PK5222	742917	206	3.8E+06	5.5E−05
235	-	-	Torque teno virus	68887	23	5.5E+06	4.2E−06
73	-	TTV	Torque teno midi virus 1	687379	137	6.9E+06	2.0E−05
505	-	-	Torque teno virus	68887	37	6.9E+06	5.3E−06
505	-	-	Small anellovirus	393049	25	6.9E+06	3.6E−06
457	-	-	Torque teno virus	68887	29	1.5E+07	1.9E−06
171	-	-	Torque teno mini virus 2	687370	18	1.2E+06	1.6E−05
159	-	TTV	Torque teno mini virus 5	687373	143	2.6E+06	5.6E−05
179	-	-	Torque teno mini virus 1	687369	17	1.8E+06	9.3E−06
193	-	-	Torque teno mini virus 2	687370	56	1.6E+06	3.6E−05
183	-	TTV	Torque teno mini virus 3	687371	139	3.0E+06	4.6E−05
156	-	TTV	Torque teno midi virus 1	687379	213	2.3E+06	9.1E−05
186	-	-	Torque teno virus 15	687354	1701	2.0E+06	8.3E−04
282	-	TTV	Torque teno midi virus 1	687379	61	1.6E+06	3.7E−05
335	-	-	Torque teno virus	68887	47	1.7E+06	2.8E−05
330	-	-	TTV-like mini virus	93678	77	1.8E+06	4.2E−05
270	-	-	Torque teno virus 8	687347	82	1.2E+06	7.1E−05
331	-	-	Torque teno midi virus 2	687380	113	1.4E+06	8.2E−05
349	-	TTV	Torque teno midi virus	432261	47	1.6E+06	2.9E−05
350	-	TTV	Torque teno mini virus 4	687372	51	3.0E+06	1.7E−05
566	-	TTV	Torque teno mini virus 4	687372	206	1.9E+06	1.1E−04
377	-	-	Torque teno mini virus 4	687372	153	6.6E+06	2.3E−05
168		TTV^c^				1.9E+05	
263	-	TTV				1.5E+06	

(a) The NCBI TaxID and name of the virus species with the highest number of hits among those viruses with BLAST hits is given.

(b) These two samples were prepared from aliquots of the same serum sample.

(c) In its deep sequencing dataset, Sample 168 had 9 reads matching TTV, just below our positive identification threshold.

We applied *in vitro* transcribed poliovirus RNA diluted into HeLa cell total RNA to the Virochip as an additional positive control to quantify Virochip sensitivity. Using the E-predict analysis tool, the lowest detectable concentration of poliovirus was 1 viral RNA per 10^5^ HeLa RNA molecules (approximately 10 polio gRNAs per cell equivalent of HeLa RNA; Figure S1).

### Deep Sequencing Analysis

A total of 130 serum samples were deep sequenced, including 7 positive controls and 123 previously undiagnosed samples. We performed deep sequencing on 34 of the serum samples using the Illumina GAII platform, generating a total of 184.6 million 65-nucleotide long paired-end reads (one flow cell lane each for four sample pools, 12.0 billion bases total, median of 3.7 million reads per sample). We sequenced 96 serum samples (pool five) on a HiSeq 2000 instrument, which provides more, longer sequences per run. The HiSeq run generated 196.4 million 97-nt sequences (one flow cell lane, 19 billion bases total; median of 1.7 million reads per sample).

**Figure 1:**
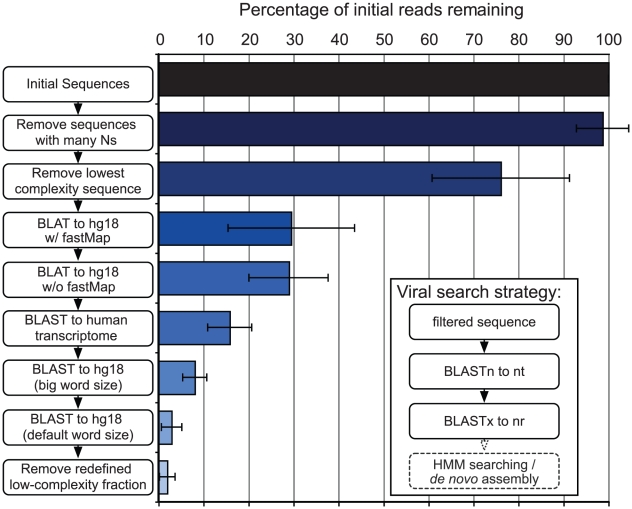
Bioinformatic filtering of deep sequencing data. Average percent remaining reads after each of the filtering steps. Low-quality and low-complexity reads are removed first, followed by iterative BLAT and BLAST comparisons to human sequence. Averages were calculated for all samples (n = 130). Inset: secondary pipeline depicting post-filtering viral searches. The dashed bubble includes future methods to improve the sensitivity of viral sequence detection.

The raw reads were first separated by barcode and analyzed as individual data sets as described in the Methods. The bioinformatic filtering process consisted of removing low complexity and low quality sequences, then filtering sequences of human origin (Figure 1). After the filtering steps, an average of 1.9% of the initial reads remained, with an absolute average of 60,000 reads remaining per sample (Figure 1 and Table 1). A few of the barcode datasets appeared to have a larger non-human fraction. Upon further inspection, the non-human components were accounted for by known library preparation contaminants, such as *E. coli* and *S. cerevisiae*.

### Detection of Known and Divergent Viruses

The reads remaining after filtering were then compared to sequences in the NCBI non-redundant nucleotide and protein databases using BLASTn and BLASTx respectively. Virus-derived sequences were detected in all 7 positive control samples and in 45/123 (37%) of previously negative serum samples (Table 1). In 78/123 (63%) samples, we were unable to identify virus sequence by our detection criteria (Methods).

We recovered virus sequences matching the expected viral genomes in all of the positive control samples. The fraction of viral sequences in the controls spanned 4 orders of magnitude, from 0.002% to 2.8% of total reads. The two HAV positive control samples (#401) were aliquots of the same serum sample and were processed and analyzed independently. The fraction of viral reads in the duplicates was within 4-fold (0.4% and 1.2%). This demonstrates that our library preparation, sequencing, and bioinformatics pipeline is capable of reproducibly detecting evidence of clinically relevant infections.

In addition to the controls, two non-control samples contained evidence of RNA virus sequence. Both samples had reads deriving from GB Virus C (GBV-C, also known as Hepatitis G Virus) and were essentially identical to GBV-C database sequences. We detected no sequences that best aligned to dsRNA viruses or to retroviruses (except for human endogenous retrovirus and contaminating MLV RT-derived sequences, see Methods).

**Figure 2:**
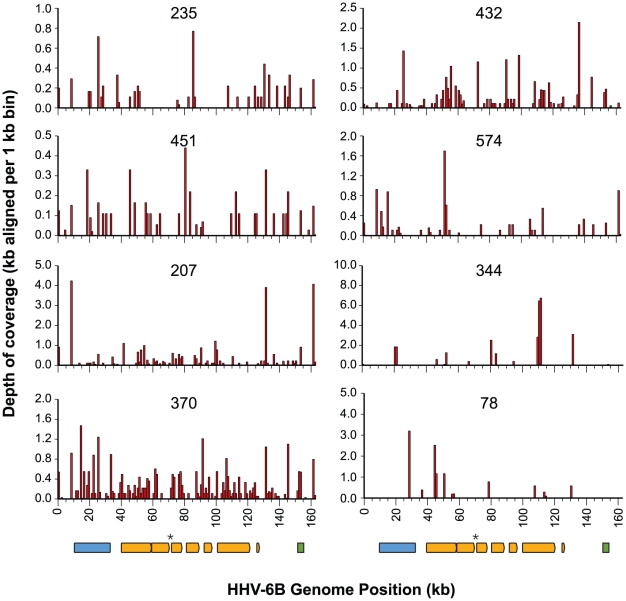
HHV-6B genome coverage in positive samples. Histograms of HHV-6B genome coverage generated by aligning reads with minimum 90% identity over the total read length to the genome. The depth of sequence coverage was calculated as the total Kb of aligned sequence per 1 Kb bin over the HHV-6B reference genome. Genome track representation adapted from Dominguez *et al* [65]. The blue box represents conserved genes across the betaherpesvirus subfamily, the orange boxes represent core genes across the herpesvirus family, the green box represents the late structural genes (gp82-105), and the asterisk denotes the origin of lytic gene replication. Inset text for each histogram is the sample code. Coverage is shown for samples with greater than 80 HHV-6 reads.

Human Herpesvirus 6 (HHV-6) sequence was detected in 13/123 previously negative samples (10.6%). The HHV-6 positive samples had an average normalized read count of 145 HHV-6 reads per sample (range: 24–411), representing 0.002% to 0.02% of the datasets (Table 1), and all of these reads possessed high sequence identity to the HHV-6B reference genome sequence (gi: 9633069). We generated alignments to the reference genome to investigate the depth and genomic position of the sequence coverage across the HHV-6 genome (Figure 2). Although the reads only constitute a relatively small fraction of each dataset, there is coverage across the entire genome and over many genes in most of the HHV-6 positive samples.

In addition to HHV-6, we detected Human Herpesvirus 4 (HHV-4, also known as Epstein Barr Virus) sequences in one sample. As with HHV-6, The HHV-4 sequences were virtually identical to previously reported sequences. One sample also contained reads similar to another dsDNA virus, African Swine Fever Virus (ASFV), which has been previously detected in human serum [42]. In this case, the reads best matched ASFV capsid sequences and were relatively divergent (47–51% amino acid identity; no similarity to non-ASFV sequences by BLASTx). Attempts to recover additional ASFV sequence by PCR were unsuccessful.

We also identified sequences derived from single-stranded DNA viruses in some samples. In one sample we detected Parvovirus B19-derived reads with high identity to database sequences. Sequences related to various members of the *Anelloviridae* virus family (TTVs) were detected in 21 (17%) samples. This frequency of detection is within the range reported previously for human serum [43], [44]. The TTV sequences ranged from 40–97% amino acid identity to their closest database matches. We did not pursue these sequences further, because TTVs are known to form a divergent family of viruses and are commonly detected in apparently healthy individuals.

Sequences similar to members of the *Circoviridae* family of ssDNA viruses were detected in 13/123 samples (10.6%). All of the sequences aligned to circovirus or circovirus-like replicase protein sequences. The alignments ranged from 36–84% amino acid identity, and appeared to derive from the replicase genes from multiple related species (Table 1). Circovirus-like replicase sequences have been detected in human stool, animals, and environmental samples [45]–[48]. We detected a range of 12 to 205 circovirus-like reads per positive sample (Table 1). The low sequence coverage prohibited complete genome sequence assembly but informed sequence-specific primer design, from which we were often able to recover larger continuous regions of the replicase genes by PCR and Sanger sequencing (GenBank accessions JF781513, JN837698, and see Table S2).

**Figure 3:**
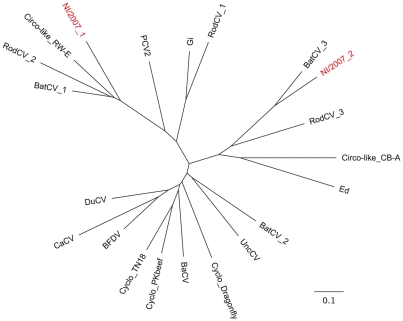
Circovirus-like NI sequence coverage and phylogeny. Phylogenetic neighbor-joining tree of amino acid sequences showing the relationship between Circovirus-like NI rep sequences (red) and 19 representative replicase sequences. Abbreviations: CV, circovirus, Ba, Barbel, Bat, Bat ZS/Yunnan-China/2009, BFDV, beak and feather disease virus, Ca, Canary Circo-like Circovirus-like genome, Cyclo, cyclovirus, PKbeef, PKbeef23/PAK/2009, Du, Muscovy duck, Ed, *Entamoeba dispar*, Gi, *Giardia intestinalis*, PCV2, Porcine circovirus 2, RodCV, Rodent stool-associated circular genome virus, UncCV, uncultured circovirus. For a full list of sequences and accession numbers, see Table S2.

We termed the extended replicase-like sequences Circovirus-like NI/2007 1–3 (Cvl-NI 1–3), and compared them to a representative set of other replicase sequences (Figure 3). The Cvl-NI-1 sequence is most closely related to Circovirus-like virus RW-E (gi: 254688530), a circular single-stranded DNA virus previously found in reclaimed water samples in Florida [45]. The Cvl-NI-2 sequence is most closely related to a replicase sequence recovered from bat feces in Yunnan Province, China (gi: 342356307) [48]. The Cvl-NI-3 sequence did not overlap with the other sequences enough to be included in the phylogenetic analysis, but was most similar to Circovirus-like CB-A, a circovirus-like genome identified in a Chesapeake Bay environmental sample (gi: 229562105) [45].

A subset of the positive samples (Table 1) contained sequences from more than one virus, which may be evidence of co-infection. Almost all of the cases with multiple viruses involved TTV-derived sequences along with HHV-6, DENV-2, or circovirus-like sequences (samples 282, 235, 183, 270, 350, and 377). Two samples contained circovirus-like sequences with ASFV-like (sample 315) or GBV-C sequences (sample 387).

## Discussion

In this study, we examined the virus diversity in serum samples from Nicaraguan children with unknown acute febrile illness. We performed Virochip microarray and deep sequencing analyses on 7 positive control and 123 undiagnosed samples. Both of these methods succeeded in detecting the expected virus in the positive control samples. Virochip analysis produced putative viral hits in 10/123 (8%) of the previously negative samples, whereas deep sequencing revealed virus or virus-like sequences in 45/123 (37%). This study demonstrates the utility of these metagenomic strategies to detect virus sequence in multiple human serum samples and is the first to utilize second-generation sequencing to simultaneously investigate many cases of acute unknown tropical illness.

Monitoring the emergence and spread of novel human pathogens in tropical regions is a central public health concern. Metagenomic analysis enables more systemic viral detection of both known and novel viral pathogens [42] and can be employed as diagnostic supplements to pathogen detection as part of public health monitoring systems and epidemiologic surveys [9]–[12], [15]–[17], [19], [21], [23]. Despite the headway, metagenomic virus detection studies will have to confront several remaining difficulties concerning diagnostic accuracy. Foremost concerns include enhancing the sensitivity and specificity of deep sequencing-based diagnostic methods and re-evaluating the evidence for disease causality in light of increasingly sensitive nucleic acid detection and pathogen discovery methods. The former will require improved strategies to biochemically enrich and computationally identify viral sequences while reducing host background sequences. The latter will require a cautious reconsideration of criteria used to establish causal links between microbes and disease, as well as extensive case-by-case follow-up studies employing classical laboratory methods, such as serological analysis and cell culture amplification. It is important to highlight that observing viral sequence in sequencing data is insufficient to establish the role of a virus in disease causality. Like other detection strategies, deep sequencing will serve to inform secondary tests, including seroconversion assays, further nucleic acid testing, cell culture amplification, and additional investigations into plausible disease mechanisms.

We detected virus sequence at concentrations as low as ∼2 in 10^6^ reads. Virus sequence detected in a clinical sample at vanishingly low copy numbers may reflect several possible host-microbe scenarios. The sequence detected may be that of a pathogenic virus capable of causing illness at low copy number or through indirect effects, a ubiquitous non-disease causing microbe, a virus outside of its primary replication site, low-level contamination, an artifact of sample collection timing/processing, or remains of incomplete immune clearance. Additional evidence must be considered in each case to define the host-microbe relationship.

In this study, we compared the performance of the Virochip and deep sequencing for detecting virus sequence in human serum. The limit of detection of the Virochip was approximately one part in 10^5^ for the poliovirus controls, for which there are microarray probes with perfect sequence complementarity (Figure S1). The sensitivity of deep sequencing is limited by the number of reads generated per sample, or read depth. In this study, we detected virus sequences down to two parts per million. Nearly every virus that was detected on the microarray was also detected by deep sequencing; additionally, in numerous samples (n = 44), sequencing revealed viruses not detected by the Virochip (Table 1). There were two instances where Virochip analysis identified a virus (TTV) that was not detected by deep sequencing (Table 1). Deep sequencing, therefore, is a superior method for novel virus discovery, because it is more sensitive and provides more conclusive genotypic information than the Virochip. Nevertheless, the Virochip is a relatively fast and inexpensive method that is best applied to samples with expected virus copy numbers present at levels greater than 1 in 10^5^ host sequences.

We were unable to detect a virus in two thirds of the 123 dengue-like illness samples. These results could reflect true negative status, which would result from a non-viral infection, illness due to non-infectious agent, or complete immunologic clearance. Alternatively, the negative results could reflect failures in our diagnostic approaches due to imperfect sensitivity, unsatisfactory sample preparation, improper sample type, or failure to recognize highly divergent viral sequences. The presence of sequences that lack even remote similarities to known species also highlights the need for further development of *de novo* assembly methods for metagenomic data. Assembled data, increased depth, and enhanced sequenced comparison methods should enable more sensitive detection of divergent viruses in metagenomic samples.

Determining the etiology of human diseases with symptoms that overlap with dengue-like illness is important for understanding the full spectrum of emerging or previously uncharacterized pathogens in tropical populations. In this study, 10% of acute serum samples negative for dengue virus from cases of pediatric dengue-like illness were positive for HHV-6. Primary HHV-6 infection causes undifferentiated febrile illness and *exanthem subitum* (*roseola infantum* or sixth disease), an acute illness with high fever and rash that typically resolves in three to seven days [49]. *Exanthem subitum* is a common disease of infants worldwide, and HHV-6 infection most frequently occurs between 6 and 12 months of age [50], with seropositivity estimates of >95% in adult populations in developed countries [51]. The HHV-6 positive patients in this study were between 7–12 months old, and presented with fever and rash (Table S3). We detected multiple kilobases of HHV-6 sequence in each positive sample, with sequence deriving from multiple viral genomic regions (Figure 2).

After acute infection, HHV-6 can latently persist in the host quiescently, with no production of infectious virions or with low levels of viral replication. Latency is believed to endure in several cell types, including monocytes and bone marrow progenitor cells [52], [53], and may undergo chromosomal integration that can be vertically transmitted [54]. The confounding effects of chromosomal integration make differentiating between active and latent HHV-6 infections difficult when detecting HHV-6 sequence in serum DNA [55], [56]. A previous study detected integrated HHV-6 genomic sequence in ∼1% of healthy blood samples [57]. Since detection of HHV-6 nucleic acid in serum alone does not prove active viral infection, we cannot definitively confirm that the HHV-6 sequences in these samples were not derived from the vertical transmission of chromosomally integrated virus. However, the clinical, epidemiological, and virus sequence data suggest HHV-6 may be the etiologic agent in these febrile illness cases.

Primary HHV-6 infection is a major cause (∼20%) of infant hospitalizations in the United States [58], a clinical burden likely shared throughout the tropical world given similar seroprevalence rates [59]. The results of this study illustrate the importance of administering HHV-6 diagnostic tests to cases of suspected dengue-like illness in infants from dengue-endemic regions to differentiate between cases of *exanthem subitum*, a ubiquitous self-limiting childhood illness, and dengue fever, which carries a greater risk of severe clinical complications and death.

Similarly, the one sample positive for Parvovirus B19 sequence may be a case of acute infection with a commonly acquired childhood virus. Parvovirus B19 can manifest as *erythema infectiosum* (fifth disease), a condition associated with characteristic “slapped cheek” rash [60]. Infection can also be subclinical or result in mild nonspecific symptoms. It is possible that Parvovirus B19 infection caused the symptoms in this case (Table S3), though as with HHV-6, the identification of viral sequences does not definitively demonstrate causality.

Epstein Barr Virus (HHV-4) sequences were found in the serum of one patient who presented with relatively severe symptoms, and died during hospitalization (Table S3). HHV-4 infection is a nearly universal occurrence in the first two decades of life [61], [62]. Primary infection in adolescents or adults can manifest as infectious mononucleosis, and chronic infection is associated with various malignancies later in life. Primary infection during childhood, however, is usually asymptomatic or produces only mild symptoms. It is not clear that HHV-4 infection or HHV-4 alone caused the illness in this case.

In addition to the viruses for which a plausible disease association exists, many samples contained sequences from viruses with no well-established link to human disease. These included the two samples positive for GBV-C and those containing ASFV-like, TTV-like, and circovirus-like sequences.

The *Circoviridae* family is an extraordinarily diverse group of small, single-stranded circular DNA viruses that includes cycloviruses (genus *Cyclovirus*) and circoviruses (genus *Circovirus*), which are commonly detected in human stool and blood, and also in environmental samples [43]–[48]. Some circovirus species, such as beak and feather disease virus and porcine circovirus 2, have been associated with disease in bird and pig hosts, respectively, but the pathogenic potential of circoviruses in humans remains unconfirmed [63], [64]. The circovirus-like sequences reported here were detected in nucleic acid libraries prepared from acute human serum and were most closely related to circovirus-like viruses (Figure 3), which were first reported in environmental samples and in bats [45], [48]. We were unsuccessful in recovering a full genome sequence corresponding to any of the circovirus-like sequences, and it has not yet been possible to prove that these sequences were not an environmental artifact introduced during sample preparation. It is also possible that these sequences derive from other organisms, such as *Giardia intestinalis* or *Entamoeba dispar*, whose genomes encode proteins that share amino acid similarity with circovirus replicase proteins (Figure 3). Furthermore, it has yet to be established whether circoviruses are capable of replicating in humans. Pending additional screening and serologic studies, the detection of circovirus-like sequences from human serum should be interpreted with caution.

Metagenomic approaches provide an effective high-throughput method to detect uncharacterized virus diversity in a tropical setting from many samples simultaneously. The findings presented in this study further our knowledge of well-characterized and previously unknown viruses present in serum collected from pediatric dengue-like illness patients and advance our understanding of the application of metagenomic approaches to human pathogen detection. Deep sequencing analysis of clinical samples holds tremendous promise as a diagnostic tool by permitting the detection of many different viruses simultaneously, including those present at low-copy numbers and of divergent origin. Major remaining barriers to high-throughput sequencing strategies becoming standard diagnostic practice include prohibitive cost, lengthy sample preparation time, and computationally intensive data analysis requirements. These challenges are magnified in resource-limited settings, such as Nicaragua, but are gradually being addressed. Industry hardware and technical advancements have steadily decreased the per-base cost of deep sequencing, and the results presented here strengthen our expectations of multiplexed sample preparation and bioinformatic data filtering within the framework of current second-generation sequencing platforms. Long-term bi-directional partnerships with developing country collaborators facilitate easier access to techniques not currently available on-site, such as deep sequencing, and are also important in providing training opportunities for local scientists and developing relevant pathogen tests and diagnostic policies.

This study expands our understanding of the virus diversity in pediatric dengue-like illness in Nicaragua and the application of genomic detection techniques in a tropical setting, findings that are particularly valuable given the pressing need for improved global emerging pathogen surveillance.

## Supporting Information

Figure S1 **Virochip sensitivity using poliovirus control RNA.** The Virochip can detect one poliovirus gRNA in a background of 10^5^ HeLa RNA molecules. Poliovirus RNA was mixed with HeLa total RNA and analyzed on the Virochip. Eighty enterovirus Virochip oligos were found to be responsive to the poliovirus RNA and the mean fold above background of the normalized intensity of these oligos is plotted. Background is defined as the normalized intensity for each oligo in the HeLa-only control sample. The top E-predict hit in the 10^−5^ to 10^−2^ samples was human enterovirus C.
(PDF)

Table S1 **This table lists the sequences of the oligonucleotides used in this study.**
(PDF)

Table S2 **This table lists the accession numbers of protein sequences used to construct the phylogenetic tree in** **Figure 3** **.**
(PDF)

Table S3 **This table displays the clinical signs recorded during hospitalization.**
(PDF)
