# Supplementary figures and images for: Virus Identification in Unknown Tropical Febrile Illness Cases Using Deep Sequencing
Source: PLoS Negl Trop Dis. 2012 Feb 7;6(2):e1485. doi: 10.1371/journal.pntd.0001485 (PMC3274504; doi:10.1371/journal.pntd.0001485)

Yozwiak *et al* Figure S1

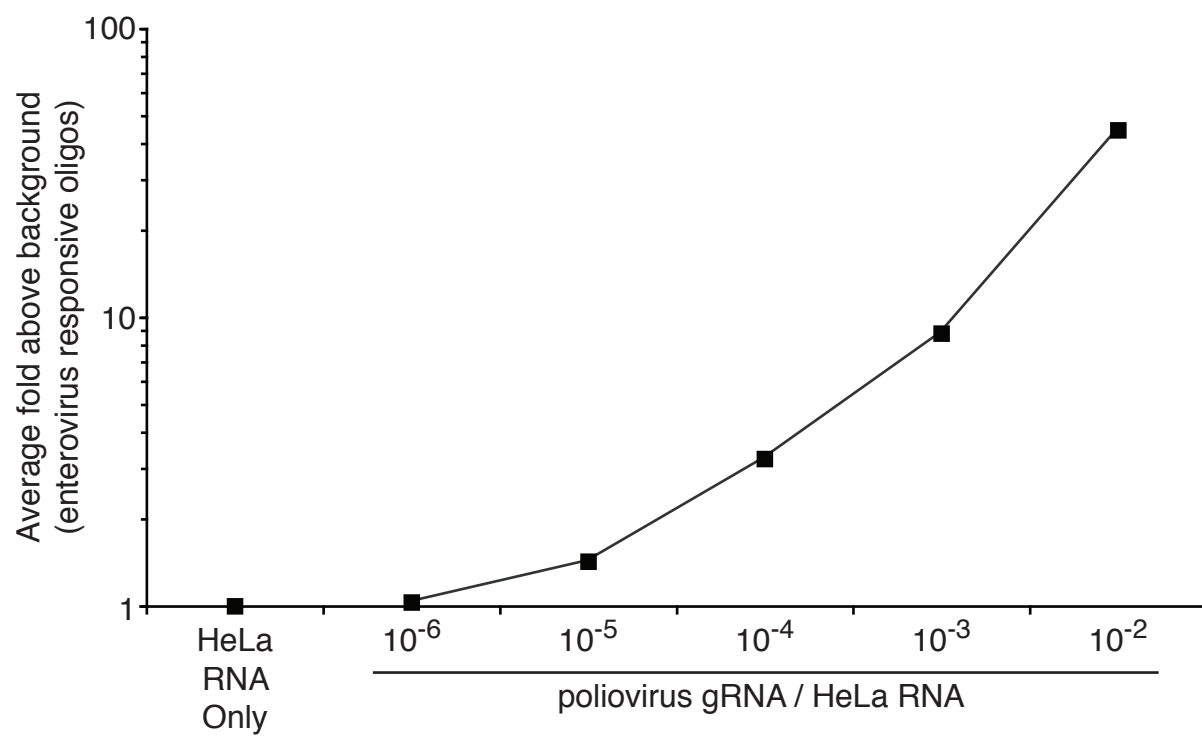

Supplement: Figure S1 — Virochip sensitivity using poliovirus control RNA. The Virochip can detect one poliovirus gRNA in a background of 105 HeLa RNA molecules. Poliovirus RNA was mixed with HeLa total RNA and analyzed on the Virochip. Eighty enterovirus Virochip oligos were found to be responsive to the poliovirus RNA and the mean fold above background of the normalized intensity of these oligos is plotted. Background is defined as the normalized intensity for each oligo in the HeLa-only control sample. The top E-predict hit in the 10−5 to 10−2 samples was human enterovirus C. (PDF) [file pntd.0001485.s001.pdf]
