# Supplementary material for: Virus Identification in Unknown Tropical Febrile Illness Cases Using Deep Sequencing
Source: PLoS Negl Trop Dis. 2012 Feb 7;6(2):e1485. doi: 10.1371/journal.pntd.0001485 (PMC3274504; doi:10.1371/journal.pntd.0001485)
Supplement: Table S3 — This table displays the clinical signs recorded during hospitalization. (PDF) [file pntd.0001485.s004.pdf]

Table S3, Clinical signs recorded during hospitalization[1]
Yozwiak *et al*

Code	Viruses identified	Sex	Age [2]	Fever	Retro-orbital pain	Headache	Dehyration	Myalgia	Arthralgia	Periorbital edema	Facial edema	Mottled skin	Distal coldness	Shock	Respiratory difficulty	Irregular respiration	Cough	Rhonchi	Crackle	Wheezing	Pneumonia	Intercoastal retractions	Abdominal tenderness	Abdonominal pain	Intermittent pain	Continuous pain	Abdominal distension	Ascites	Jaundice	Macular exanthem	Papular exanthem	Maculopapular exanthem	Erythema	Flushed face	Tourniquet test positive	Lethargy	Bleeding	Petechiae	Hemoptysis	Epistaxis	Melena	Subconjunctival hemorrhage	Hepatomegaly	Splenomegaly
187	DENV-2	M	3y	+	+	+	+	+									+						+	+	+								+	+	+		+	+						
275	DENV-2	M	4y		+	+	+	+	+	+			+	+			+						+	+		+	+	+																
282	DENV-2, TTV	F	4y		+	+	+	+	+			+	+	+									+	+	+		+	+		+														
266	DENV-2	F	11y																											+			+	+			+	+						
274	DENV-1	F	14y	+		+	+	+	+																					+														
401	HAV	F	8y																					+	+				+															
235	HHV-6, TTV	F	7m																														+	+			+	+						
451	HHV-6	M	1y	+									+				+	+												+			+	+	+		+	+						
207	HHV-6	M	9m	+													+																											
432	HHV-6	M	1y														+													+		+	+				+	+						
574	HHV-6	M	9m	+																												+	+	+										
370	HHV-6	F	1y																								+			+	+	+	+		+		+	+						
78	HHV-6	M	8m	+																													+											
131	HHV-6	M	9m																																									
183	HHV-6, TTV	F	18m							+			+														+	+								+	+		+		+			
270	HHV-6, TTV	M	1y																																								+	
344	HHV-6	F	6m														+				+																							
350	HHV-6, TTV	M	10m																																+									
438	HHV-6	M	13y																																									
382	HHV-4	M	4y				+			+	+	+	+	+	+	+						+	+	+		+	+	+	+						+	+	+	+						
387	GBV-C, Circo	M	12y			+											+													+			+	+	+								+	+
180	GBV-C	F	9y			+			+																																		+	
161	ParB19	M	5y		+												+																		+		+	+					+	
118	Circo	F	6y														+																		+		+	+						
323	Circo	F	1y														+													+			+	+										
363	Circo	F	6y	+																																	+	+					+	
371	Circo	F	11y		+	+		+	+								+													+			+		+								+	
355	Circo	F	9y																					+	+		+	+		+			+	+	+		+	+						
345	Circo	F	8y														+				+														+		+	+						
315	Circo, ASFV	F	11y		+	+		+	+								+																											
329	Circo	M	10y		+	+		+	+																																		+	
321	Circo	M	14y		+	+		+	+																												+	+					+	
375	Circo	F	9y		+	+		+	+								+																				+	+						
377	Circo, TTV	F	3y			+		+	+																												+			+				
322	Circo	F	1y														+																											
73	TTV	M	2y	+																			+		+								+										+	
505	TTV	M	14y	+		+																								+			+										+	
457	TTV	F	9y							+																				+			+	+	+		+	+					+	
171	TTV	F	14y														+																											
159	TTV	M	2y	+													+	+																			+	+					+	
179	TTV	M	18m														+																	+	+		+	+						
193	TTV	F	11m																														+										+	
156	TTV	F	6y		+	+											+																											
186	TTV	M	14y	+	+	+	+	+	+																					+			+	+	+		+	+					+	
335	TTV	F	7y														+	+	+	+	+									+			+	+	+		+	+				+	+	
330	TTV	F	8m																											+			+	+										
331	TTV	M	5y			+																																					+	
349	TTV	F	1y	+														+																										
566	TTV	M	1y																				+																					
168	TTV	M	14y	+	+	+		+	+																										+		+	+						
263	TTV	M	10y																														+	+			+	+					+	+

(1) Clinical signs recorded during hospitalization. Note that study inclusion criteria were based on reported symptoms at time of hospital admission.
(2) Age of patient at time of hospitalization. y, years; m, months.
