# Supplementary material for: Virus Identification in Unknown Tropical Febrile Illness Cases Using Deep Sequencing
Source: PLoS Negl Trop Dis. 2012 Feb 7;6(2):e1485. doi: 10.1371/journal.pntd.0001485 (PMC3274504; doi:10.1371/journal.pntd.0001485)
Supplement: Table S2 — This table lists the accession numbers of protein sequences used to construct the phylogenetic tree in Figure 3 . (PDF) [file pntd.0001485.s003.pdf]

Table S2, Protein Sequences used to construct phylogenetic tree in Figure 3.
Yozwiak et al

Abbreviation	NCBI GI	Protein	Organism
BaCV	330339439	putative replication protein	Barbel circovirus
BatCV_1	342356276	replication-associated protein	Bat circovirus ZS/Yunnan-China/2009
BatCV_2	342356282	replication-associated protein	Bat circovirus ZS/Yunnan-China/2009
BatCV_3	342356307	replication-associated protein	Bat circovirus ZS/Yunnan-China/2009
BFDV	11119205	putative replicase-associated protein	beak and feather disease virus
CaCV	18875310	replicase	Canary circovirus
Circo-like_CB-A	254688534	putative Rep protein	Circovirus-like genome CB-A
Circo-like_RW-E	254688531	putative Rep protein	Circovirus-like genome RW-E
Cyclo_Dragonfly	324309787	replication associated protein	Dragonfly cyclovirus
Cyclo_PKbeef	316989866	Rep protein	Cyclovirus PKbeef23/PAK/2009
Cyclo_TN18	290783653	replication-association protein	Cyclovirus TN18
DuCV	269979225	rep	Muscovy duck circovirus
Ed	167387593	replication-associated protein	Entamoeba dispar SAW760
Gi	6840822	putative replication-associated protein REP2	Giardia intestinalis
PCV2	222160098	ORF1	Porcine circovirus 2
RodCV_1	343196930	REP	Rodent stool-associated circular genome virus
RodCV_2	343196943	REP	Rodent stool-associated circular genome virus
RodCV_3	343196958	REP 2	Rodent stool-associated circular genome virus
UncCV	333596611	Rep	uncultured circovirus

CvI-NI-2 sequence, which is below the limit of 200 nucleotides for GenBank submission:
>Circo-like_NI_2007_Rep_2 [organism=Circovirus-like_NI/2007-2] Circovirus-like NI/2007-2 Rep protein gene, partial cds
CCGTCCACGAAGCTTAGGCGTCTTAAATGATCTCGAGAATGAATGGTGGTATGGAGATACCGGTACAGGCAAATCTAAAAAACTTTGGGAACTATAT
CCAAATCATTACGGCAAAGCACTTAATAAATGGTGGGATGGTTATAATGATGAAGATATTGTAGTCATTGAAGAAATGACCCCAGATGCTGGAT
