# Supplementary material for: Virus Identification in Unknown Tropical Febrile Illness Cases Using Deep Sequencing
Source: PLoS Negl Trop Dis. 2012 Feb 7;6(2):e1485. doi: 10.1371/journal.pntd.0001485 (PMC3274504; doi:10.1371/journal.pntd.0001485)
Supplement: Table S1 — This table lists the sequences of the oligonucleotides used in this study. (PDF) [file pntd.0001485.s002.pdf]

Table S1 - Oligonucleotides used in this study
Yozwiak et al

Primer Name	Description	Sequence (5'-3')
SolCommonBar+hex	Reverse transcription (pools 1-4)	CGCTCTTCCGATCTN<barcode>NNNNNN (a)
SolCommonBar	Library amplification (pools 1-4)	CGCTCTTCCGATCTN<barcode> (a)
SolCommon+hex	Reverse transcription (pool 5)	CGCTCTTCCGATCTNNNNNN
SolCommon	Library amplification (pool 5)	CGCTCTTCCGATCT
pr2	Illumina adapter A, short	CTACACGACGCTCTTCCGATCT
pr3	Illumina adapter A, full length	AATGATACGGCGACCACCGAGATCTACACTCTTTCCCTACACGACGCTCTTCCGATCT
pr4	Illumina adapter B (pools 1-4)	CAAGCAGAAGACGGCATACGAGATCGGTCTCGGCATTCCTGCTGAACCGCTCTTCCGATCT
pr4-bar	Barcoded illumina adapter (pool 5)	CAAGCAGAAGACGGCATACGAGAT<barcode>GTGACTGGAGTTCAGACGTGTGCTCTTCCGATCT (b)
MAMD	DENV sequence recovery (c)	AACATGATGGGRAARAGRGARAA
cFD2	DENV sequence recovery (c)	GTGTCCCAGCCGGCGGTGTCATCAGC
MDS-47	HAV sequence recovery	CTAAATTGGGGACRCAGATG
MDS-48	HAV sequence recovery	TGAGGAAAAACCTAAATGCC
Cvl-NI-1 1F	Circovirus-like rep sequence recovery (CvL-NI-1)	GGGTGAGATTGGTGCTGGTA
Cvl-NI-1 4R	Circovirus-like rep sequence recovery (CvL-NI-1)	CCTTTTTGCTCCACAAGGACT
MDS-227	Circovirus-like rep sequence recovery (CvL-NI-1)	TGAATTTACTGGTCAGGTGGAG
MDS-230	Circovirus-like rep sequence recovery (CvL-NI-1)	GCTTCCTGGTTCAAATCTTGG
MDS-267	Circovirus-like rep sequence recovery (CvL-NI-2)	CGAAGCTTAGGCGTCTTAAATG
MDS-270	Circovirus-like rep sequence recovery (CvL-NI-2)	GCATCTGGGGTCATTTCTTC
MDS-219	Circovirus-like rep sequence recovery (CvL-NI-3)	GCTGCTAAAGGAACGACTGAAC
MDS-221	Circovirus-like rep sequence recovery (CvL-NI-3)	GTTTCCAGTCGGGCCATAG

(a) Pool 1-4 barcodes:
AAA, ACC, AGG, ATT, CAC, CCA, CGT, CTG, GAG, GCT, GGA, GTC, TAT, TCG, TGC, TTA, AAAA, AACC, AAGG, AATT, ATAT, ATTA
(b) Pool 5 barcodes:
GGTCTAA, ACAGAAG, TTGACCT, CACTGGA, AAGATCC, CCTTCAA, TGAGGTA, GTCCAAC, AACACCT, CCACTAA, GGTTCTT, TTGGTTG,
TCCGGTT, GTACTAG, CATTGAC, AGGAATC, AATTCCG, TCAATCC, TAACCAC, TCAGACT, CTAGCGA, TTGTCAC, GGCACTA, TTACACC,
GGATTCC, CCGAACT, TCTTGTC, CGCTATG, ATCGACC, GTCATTG, GGTAATG, TGGCAAG, TGAAGAC, TAAGTGC, AACTTGC, AATCGTC,
TAAGGAG, ATAGGAC, ACTACTG, CGTTACC, GGTACAC, GGACAAT, TTAGAGG, ATGCACT, TATCAGG, GCAACAT, GTTGGTC, TACCGAA,
GTTGAAG, GTGAACC, AAGTGGT, TCTAGGT, GCATGAA, CAACGTA, TTCTTCC, AACAGTG, TTACGTG, CCAAGTC, CTGAGTG, TAGCCTA,
GATGCCA, TACAAGC, TAGGCGT, GAACCTT, TTCAACG, TGGTAGT, GAAGAGT, CAGTTCA, CAGGCAA, GATATGG, ACTCAGA, CACTCTT,
GCCTTAT, CAAGCCT, TAGACAG, CAATACG, TACCTTC, AAGGCTC, TGAGCAT, TATTCGC, TCCATAG, CTAATGG, TGGCTCA, CTTCATG,
AGACCGT, GAGTTAC, AGTGCTA, AACCTAG, AAGCAAC, AGATGTG, CGTTGTA, ACTGGCT, ATCTGCA, ATTCCAC, GACCATA, AGAACCA
(c) Primers from: Scaramozzino et al. J Clin Microbiol (2001) vol. 39 (5) pp. 1922-7
